# “Peer with a P versus a p”: A mixed-methods study of peer support training, service delivery, and supervision across global contexts

**DOI:** 10.1371/journal.pmen.0000447

**Published:** 2026-01-12

**Authors:** Ernesto Isaac Lara, Laura Bond, Kathryn O’Neill, Emily Ruiz, Vikram Patel

**Affiliations:** Department of Global Health & Social Medicine, Harvard Medical School, Boston, Massachusetts, United States of America; James Cook University - Singapore Campus, SINGAPORE

## Abstract

Peer support services in which people with lived experience provide non-clinical, mutualistic support are effective in improving health outcomes for people with serious mental health challenges. Despite its demonstrated effectiveness, there is limited research on peer supporters’ experiences with training, service delivery, and supervision across diverse global contexts. This explanatory mixed methods study explored these perspectives through a survey (N = 101), key informant interviews (N = 13), and focus group discussions (N = 14). Quantitative data from the survey was summarized descriptively, and qualitative data were analyzed using thematic content analysis. Participants reported high use of peer-specific competencies including sharing lived experience (89.1%), collaboration and care (81.2%), and communication (78.2%). Themes emerging from qualitative data emphasized role clarity, collaboration with non-peers, accessible training, and peer supervision. Challenges identified included stigma, inequitable compensation, limited career pathways, and inconsistent training quality. Many participants preferred peer-led or co-supervision models. In contexts without formalized peer support infrastructures, grassroots and faith-based organizations played a critical role in delivering peer support services. These findings highlight common foundations and context-specific nuances necessary for strengthening peer support across contexts, including standardized training competencies, sustainable and inclusive training, equitable compensation, and peer supervision models.

## Introduction

Serious mental health challenges, more commonly known in clinical settings as “severe mental illnesses”, affect nearly 14.6 million (5.6%) of the US adult population, and nearly one billion people worldwide [1,2]. These challenges refer to psychiatric diagnoses such as bipolar disorder, schizophrenia, post-traumatic stress disorder, and additional conditions that significantly impair daily function, and overall quality of life [3,4]. Individuals living with serious mental health challenges often face adverse life outcomes, including violence, abuse in care settings, and comorbidities [5–7]. Despite the significant impairment these challenges have on people living with such conditions, global provider shortages, care gaps, and additional factors hinder these communities’ ability to seek care [8,9]. Within the United States, nearly 3 in 10 adults (4.3 million people) with serious mental health challenges did not receive treatment, attributed to lack of knowledge of how and where to receive support, and inability to identify a preferred service provider [2]. In addition to lack of knowledge, social factors including stigma further prevent people with serious mental health challenges from seeking care [10,11]. Community members (i.e., peers) play a significant and powerful role in combatting stigma, promoting mental health, and educating others on options for support in their local community [12,13].

Given their direct involvement within the community and their organic relationships, peers are uniquely positioned to effectively bridge often unserved communities with mental health support. Peer support services leverage this naturally reciprocal relationship by deploying peers with lived or living experience of recovery to provide support to others during their recovery journey. Peer support encompasses a broad range of skills and services, including resource sharing, active listening, recovery planning, sharing lived experiences to inspire hope, and more [14,15]. Numerous studies have demonstrated the effectiveness of peer support in improving clinical, psychosocial, and recovery-oriented health outcomes [16–20].

The United States has made significant strides in developing and growing the peer support workforce within the last decade. Through the Substance Abuse and Mental Health Services Administration (SAMHSA), peer support national guidelines and suggested certification standards have provided guidance for state-level implementation of peer support trainings [14]. Additionally, organizations such as the National Association of Peer Supporters, Center for Addiction and Recovery Services, and Technical Support Centers across the nation have enabled the rapid expansion of peer support across the country as a Medicaid-billable provider type. Despite these advancements, multiple barriers such as fragmented implementation, lack of national regulation and standards, and disparities in training, supervision, and compensation hinder further scaling and expansion efforts.

Peer support services, while often perceived as relatively “new” within the United States, emerged from a broader movement for human rights and equitable mental health services, centering the collective liberation of people with lived experience (also referred to as “consumers”, “ex-patients”, and “psychiatric survivors”). Building upon these foundational values, peer support has been increasingly adopted across global contexts, with countries such as Canada, Australia, and Denmark making notable strides in building robust and sustainable peer support infrastructures [21–24]. Through findings from a recent systematic review completed by our team, we found that current research often highlights nations with robust and developed peer support workforces, such as the United States, Canda, Australia, and Denmark [25]. In global contexts where peer support service infrastructures may be absent, peer support exists in voluntary or ad-hoc capacities in which community members, through shared affinity, connect and provide mutual support. Examples include professionalized peer supporters in China; volunteer networks in South Korea; and developing initiatives in Germany, Israel, India, Tanzania, and Uganda aimed at expanding care access and reducing stigma [26–28]. Additional ongoing global collaborations, such as the EMPOWER Peer Support Initiative at Harvard Medical School and an initiative from Yale to expand peer support in Brazil also contribute to strengthen the global peer workforce (NCT05842889).

Understanding the nuances in how peer support is conceptualized, implemented, and sustained across global contexts, formally and informally, is critical in building the peer workforce globally. The current field of peer-reviewed literature is often limited to peer support in countries with robust peer support infrastructures, and/or trials conducted within a controlled environment (i.e. RCTs) necessitating further research from naturalistic settings where peer support is more informalized. Research that centers the real-world, on the ground experiences of peer supporters, rather than within a controlled environment, not only informs the broader field but also enables the adaptation of peer support programs that are attuned to the local culture and needs across global settings. Aligned with this mission, this study aimed to expand upon the field to explore the perspectives and opinions of peer supporters from across the globe. While the existing literature has affirmed the effectiveness of peer support across different global populations, little research has compared perceptions and implementation elements of peer support programs across global contexts. We aimed to address this gap by answering the following research questions: (1) What are the experiences across global settings regarding peer support training, service delivery, and supervision; and relatedly (2) how can peer supporters’ experiences inform improvements to peer support infrastructures, including certification requirements, training standards, supervision practices, and implementation protocols.

## Methods

This study uses an explanatory mixed method design to describe peer supporters’ experiences with training, supervision, and service delivery across diverse settings. We used a survey to gather descriptive information about peer supporters’ experiences delivering support to individuals with serious mental health challenges and their experiences with training, service delivery, and supervision. We then conducted key informant interviews (KIIs) and focus group discussions (FDGs) to gain further insight into the nuances of peer support, explore differences across settings, and explain the data that we found in the survey.

### Ethics statement

The Office of Regulatory Affairs and Research Compliance at Harvard University reviewed and approved the study. Written consent was obtained electronically from all participants prior to enrollment.

### Sampling and recruitment

Survey participants were recruited using convenience and snowball sampling approaches via email listservs, word of mouth, and LinkedIn advertising. The research team also utilized an expert advisory board and a global partner, aves Mental Health (formally known as the Global Mental Health Peer Network), to share recruitment materials. Prior to initiating the survey, survey participants completed a brief eligibility questionnaire indicating that they were over 18 years of age and had previous or current experience as a peer supporter for individuals experiencing mental health challenges. Consent was received electronically using a Qualtrics form. Survey participants were asked to indicate their interest in participating in a FGD and were recruited if they indicated “yes”. We stratified FGDs by geography – aiming for two FGDs with participants outside of the United States, two with participants inside of the United States, and two mixed.

We used a convenience sampling approach to select participants for KIIs with expertise in managing and/or delivering peer support interventions who served on the project advisory board and representing diverse geographic contexts.

### Data collection, measures and management

The survey was informed by findings from a systematic review that was also conducted by our team; see supplemental information for the full survey (S1 Text) [25]. As the systematic review was limited to peer support services that were included in randomized controlled trials, we used the survey and other formative research to understand the implementation elements of peer support training, services, and supervision outside of what is written about in peer-reviewed research. The questions in the survey were intended to build upon and provide further context for the findings from the systematic review by capturing the on-the-ground experiences of peer supporters. For example, during the data extraction phase of the systematic review, a taxonomy was created by thematically coding the competencies described in each of the included studies. The themes from this taxonomy, captured in codes, were used as response options when asking survey participants about the skills and techniques used within their peer support services. The survey was available in English and Spanish and available on the Qualtrics platform hosted by Harvard Medical School, collecting responses from August 2024 to March 2025. The survey included questions regarding experiences with training, supervision, and service delivery. Survey questions had varied response options, including Likert-Scale, text entry, and options to check all relevant boxes. See supplemental material one (S1 Text) for the full survey.

We conducted the KIIs and FGDs using semi-structured interview guides with open-ended questions and potential prompts. The semi-structured guides were informed and refined by the survey responses and intended to expand on findings we wanted more clarification or expansion upon. Both KIIs and FGDs were conducted using the Harvard-hosted Zoom platform. The KIIs lasted 60 minutes and covered topics such as the role of peer supporters and individuals with lived experienced in organizations, the peer support landscape in the participants’ country context or state within the US, competencies and skills required for peer support work, and the importance of appropriate language when discussing peer support work and developing trainings to deliver peer support. The FGDs lasted 90 minutes and covered topics such as the participants’ current work, the key components of successful peer support trainings, strengths and challenges of trainings, key considerations for the delivery of trainings and supervision.

All KIIs and FGDs were conducted by trained members of the research team with advanced degrees and prior training in qualitative data collection. For each FGD, two members of the research team joined to facilitate adequate tech support for participants and allow one member of the research team to focus entirely on leading the discussion. KIIs were conducted from August 2024 to November 2024, and FGDs were conducted from November 2024 to February 2025. A transcript from each KII and FGD was generated, cleaned, and de-identified by the research team prior to beginning data analysis.

Participant data, including consent forms, survey responses, and KII and FGD transcripts were securely stored using a shared Microsoft OneDrive folder only the research team had access to, on an as-needed basis. All data containing identifiers, such as name and other personal information, were de-identified prior to data analysis.

### Data analysis

We used descriptive statistics to summarize survey results and thematic content analysis to analyze data from KIIs, FGDs, and open-ended survey responses [29]. Thematic content analysis included both deductive and inductive approaches, capturing themes from previous findings and peer support competency frameworks, and themes arising from the data itself. Thus, part of the codebook was developed prior to data collection, and some codes were generated deductively based on findings from the quantitative survey data and the SAMHSA peer support competencies [14]. We developed our codebook guided by the Boyatzis approach, which includes a three-level codebook with code definitions, inclusion and exclusion criteria, and examples of the code being used in the data [30]. Using this approach enabled a more reliable coding system with less risk of bias and higher inter-coder agreement.

Three members of the research team conducted thematic content analysis and applied the same codebook to both KII and FGD transcripts. First, the research team undertook an open coding process in which each member of the coding team read transcripts in-depth, taking notes and making memos of emerging themes and patterns. Next, the team met to draft an initial codebook based on the existing deductively-generated codes and new inductive codes based on themes identified during open coding. The codebook was tested and refined over a period of several rounds of coding until all three members of the research team found that the codebook was sufficient. We used MAXQDA software to test the inter-coder agreement of each pair coders [31]. The three researchers achieved between 84–88% inter-coder agreement. After establishing inter-coder agreement, the remaining transcripts were coded while members of the research team met regularly to discuss emerging patterns and any challenges with coding certain parts of the transcripts. The PI was planned to be consulted if any discrepancy occurred, or when needed, however, no such occurrences came up during this process.

## Results

### Sample demographics

The total number of eligible survey participants was 101. 57 participants were from the United States, and the remaining 33 participants were from one of fourteen countries (N = 101). The average age of all participants was 45 (24–78, 14.0). Most participants indicated they were either cisgender male (n = 35) or female (n = 45), and were heterosexual (n = 74). Nearly two-thirds of participants identified as white or Caucasian (n = 66), and only ten participants indicated they were of Hispanic or Latino origin. In terms of the highest level of education completed, 38 participants indicated a bachelor’s degree, 18 indicated an associates or technical degree, and 14 indicated some college, but no degree. The average number of years participants served as a peer supporter was 7.8 years (n = 98, 1–33, 7.4). See supplemental material two (S1 Data) for survey participants’ demographics.

FGD participants were stratified by geography resulting in two FGDs comprised of participants based in the United States, one FGD with participants outside of the United States, and one FGD with participants from both stratification groups, due to participant availability and scheduling. A total of 14 participants took part in an FGD. 7 participants indicated they were from the United States, 6 indicated they were from outside of the United States, and 1 participant did not provide a response. The average age of FGD participants was 42.1 (24–63, 13.3). Half of the participants were cisgender male (n = 7) and heterosexual (n = 7). Most participants were white or Caucasian (n = 6), and no participants indicated they were of Hispanic or Latino origin (N = 14). A majority of participants indicated the highest level of education they completed was a bachelor’s degree. The average number of years participants served as a peer supporter was 4.9 years (n = 13, 2–15, 3.6); one participant’s response was removed due to inconsistencies. See supplemental material three (S2 Data) for focus group discussion participants’ demographics.

13 participants were recruited to take part in the KIIs, 5 from the United States and 1 participant each from Egypt, South Africa, Australia, Nigeria, Denmark, Kenya, Israel, or Singapore.

### Survey findings

Table 1 presents survey data on peer supporters’ experiences with training, certification, service delivery, and supervision.

**Table 1:** Survey Data on Peer Support Training, Certification, and Supervision.

Variable	Total Sample	US Participants	Non-US Participants
	(N = 101)	(n = 59)	(n = 42)
**Training Data**			
***Modality (n, %)***			
In-Person	51 (50.1)	29 (28.7)	22 (21.8)
Virtual or Online	29 (28.7)	21 (20.8)	8 (7.9)
Hybrid	21 (20.8)	9 (8.9)	12 (11.9)
***Cost (n, %)***			
*Was there a cost associated with training*			
Yes	34 (33.7)	23 (22.8)	11 (10.9)
*Was any financial assistance, such as scholarships, available?*			
Yes	18 (53)	12 (35.3)	6 (14.6)
No	16 (47.1)	11 (32.6)	5 (14.7)
No	67 (66.3)	36 (35.6)	31 (30.7)
**Certification Data**			
***Certification Status (n, %)***			
Certified	82 (81.2)	52 (51.5)	30 (29.7)
Not certified	19 (18.8)	7 (6.9)	12 (11.9)
***Requirements for certification (n, %)***			
Training	78 (77.2)	50 (49.5)	28 (27.7)
Standard Examination	46 (45.5)	39 (38.6)	7 (6.9)
Internship/Practice	23 (22.8)	16 (15.8)	7 (6.9)
***Average hours of training required for certification (Range, SD)***	71.4 (9-120, 32.7)	65.4 (20-120, 28.9)	82.6 (9-120, 36.7)
***Average hours of internship/practice required for certification (Range, SD)***	164.6 (35-200, 59.8)	189.3 (40-200, 41.3)	102.7 (35-200, 55.5)
**Supervision Data**			
***Previous experience as a supervisor? (n, %)***			
Yes	48 (47.5)	33 (32.7)	15 (14.9)
Average number of years as a supervisor (Range, SD)	8.4 (0-51, 11.3)	9.4 (0-51, 12.4)	6.3 (1-27, 8.1)
*Additional training required for supervision? (n, %)*			
Yes	30 (62.5)	20 (41.7)	10 (20.8)
No	17 (35.4)	13 (27.1)	4 (8.3)
No	46 (45.5)	21 (20.8)	25 (24.8)
Does not state	7 (6.9)	5 (5)	2 (2)

Half of survey participants (50.1%) reported taking their training in-person, and a third of participants (33.7%) indicated a cost was associated with their training. Out of the participants that indicated a cost, only 53% reported that some form of financial assistance was available to them. Survey participants reported the requirements they had to meet to receive peer support certification, which included training, standard examination, and internship/practice. 77.2% of participants reported that training was a requirement for certification, 45.5% indicated standard examination, and 22.8% indicated internship were required. The average number of hours required for training were 71.4 hours, and 164.6 hours of internship/practice.

**Fig 1:**
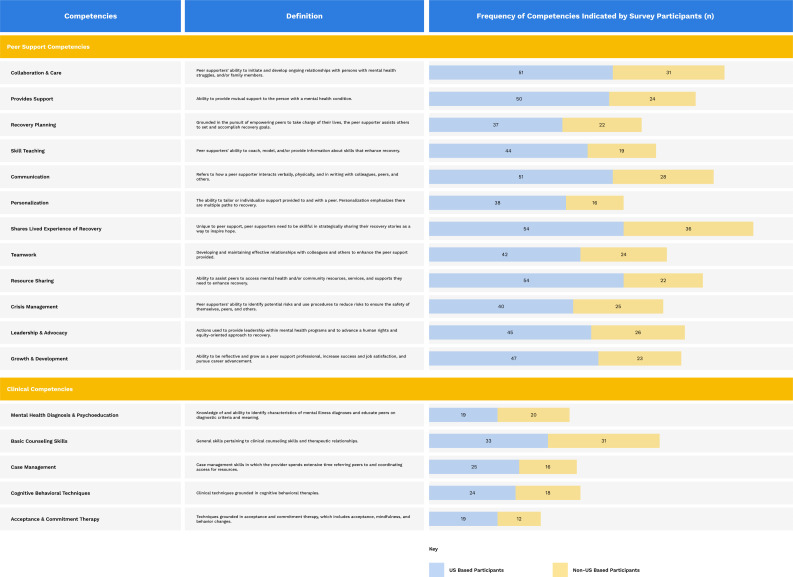
Frequencies of Competencies.

Survey participants (N = 101) were asked to indicate which peer support and clinical competencies were relevant to their service delivery. The most reported peer support competencies were sharing lived experience of recovery (89.1%, n = 90); collaboration and care (81.2%, n = 82); and communication (78.2%, n = 79). While survey participants reported using some clinical skills in practice – particularly basic counseling skills (63.4%, n = 64), cognitive behavioral techniques (41.6%, n = 42), and case management (40.6%, n = 41) - their role within peer support was more complex. See Fig 1 for the frequencies of each competency.

47.5% of survey participants indicated that they had previous experience as a supervisor to peer supporters, with an average of 8.4 years of experience as a supervisor. 62.5% of participants that reported previous experience indicated that additional training was required to become a supervisor.

### Focus group and key informant interview findings

#### Roles of the peer supporter.

Findings from our mixed method study revealed a nuanced perception of the role of peer supporters. One participant described the different ways that one can be a “peer”:

*“People can be peers to each other for lots of different reasons... They can be classmates, they can be women, they can be all sorts of things that positionally put you in a peer relationship with someone because of an affinity group that you belong to. (When) positionality is around the mental health condition, the mental health struggles, or the navigating of those systems, because similarly, you’ve done that yourself... I call that peer with a capital P. Any other kind of peer is peer with a) lowercase p.”* (Key Informant, United States)

In FGDs and KIIs, participants described the skills and techniques they be used in practice, from “using tools like the wellness recovery action plans” (Key informant, United States) and “being there to advise, not to guide” (Key informant, Kenya). For example, a KII participant from the US described how decisions are made collaboratively:

*“There might be (a peer supporter) who isn’t a fan of medication, but the person they’re serving wants to take medication. Then it’s our job to support them and figure out how they want to do that...we are not enablers of people. We are supporting people to be as independent as possible.”* (Key Informant, United States)

Lived expertise and training peer supporters on how to strategically share their lived experience was repeatedly mentioned in FGDs and KIIs. Participants emphasized the importance of “understanding worldview, and that people think very differently” (Key informant, United States) and the need to “talk about their own experiences... (but) don’t get caught up in your own experiences being the only way to feel better” (Key Informant, Denmark). Another participant stressed the importance of recognizing recovery as a personal journey that is different for everyone, stating “they say recovery isn’t linear, but everyone wants a linear story” (Focus Group Discussion Participant, United States).

Participants, particularly in non-US settings, emphasized the need to make language easily understandable: “people need information in a straight-to-the-point, easy-to-comprehend manner. And so, communication skills are important” (Key Informant, Nigeria).

Participants in KIIs and FGDs often described using these skills informally or as secondary tools, sharing concerns about the co-option and conflation of peer support with clinical or administrative roles. Some participants expressed concern about the boundaries of peer support, saying “peers shouldn’t be involved in diagnosing individuals” (Key Informant, United States), and “(peer supporters) should not be responsible for the bottom line of anything, like safety planning in a hospital” (Focus Group Discussion Participant, Mixed).

#### The ecosystem for peer support.

Many of the participants in the KIIs and FGDs discussed how they interact with clinicians during their work and the importance of clear boundaries between the clinician’s role and the role of peer supporters. Participants discussed some structural barriers that persist, particularly the power imbalance with clinicians: “I think there’s a lot of power around clinical and peer work. ‘I’m the clinician, I’m the one that does that stuff you don’t do so you need to stay in your lane’”. (Key Informant, Australia). However, other participants saw the role of the peer as complementary to clinical work: “(peer support) cannot be done in isolation. There needs to be a coordinator, an overseer, because these are people who are recovering. (They are) taking medication and they may need a clinician” (Focus Group Discussion Participant, Global).

Generally, participants expressed the value of peer-led organizations being independent but complimentary to existing systems to maintain authenticity and a recovery-focused culture. For example:

*“A huge suggestion right now is to contract with peer-run organizations so that they can still provide those services and have that available to people. But it’s also in its own bubble, away from all the clinical influence. Not saying that they can’t, you know, work together, go to lunch things like that, but it gives them that administrative bubble so that it’s a little bit more protective of the peer support role in the peer support space.”* (Key Informant, United States)

Participants in both the US and global KIIs and FGDs highlighted the vital role that community organizations play in developing, supporting, and sustaining peer support within mental health systems. In some lower-resource settings where the role of a peer supporter has not yet been formalized, faith-based institutions and grassroots initiatives fill gaps in mental health care services with activities like “hosting international training sessions” (Key Informant, Egypt) and “creating podcasts, organizing conferences, etc.” (Key Informant, Israel). Participants also expressed how community organizations may address lack of resources that make it “really hard to implement any project or program to support people in discriminated communities” (Focus Group Discussion Participant, Mixed). The role of peer supporters’ own identity in addressing such gaps among their community was also explored: “I’m queer, and they might be the only queer or trans kid on the unit and getting support that way and just knowing that they’re not alone there.” (Focus Group Discussion Participant, Mixed).

#### Training.

In the FDGs and KIIs, many themes emerged highlighting the need for flexibility and accessibility when it comes to training settings. Participants emphasized that virtual trainings are more cost effective and can increase reach “because they save participants time and money...it allows them to participate without leaving their countries, even if they can only dedicate a few hours per week.” (Key Informant, Egypt). However, virtual training can also limit engagement, experiential learning, and interpersonal connection, causing some participants to prefer in-person or hybrid training. One participant expressed that they benefit a lot more (from) an in-person training... “It’s easier to just focus and get that training done and out of the way.” (Key Informant, Nigeria).

Some participants also had concerns about representation, inclusion, and translation, in visual content and curriculum language of virtual trainings. One participant shared that trainings should not be showing “all white people, and all thin people, and able-bodied people.” (Key Informant, United States).

Participants expressed that current trainings are too brief and insufficient to prepare individuals for the role of a peer supporter. Participants also suggested that ongoing and additional training could be helpful for those entering the peer support workforce.

*“It’s too short. It’s something they get when they start in this position and then they don’t get it again. The peer support workers are always asking, when will I get more training and is this it?... When you get this training, you still have to go out there and practice... Training should be longer, and it should be like something that we keep practicing instead of just thinking that two months of training will make you a great peer support worker forever.”* (Key Informant, Denmark)

#### Certification.

In the FDG and KII discussions, many participants highlighted concerns related to certification. While most states have certification programs with required training components, there is significant variation across states, leading to a lack of consistency. In some high-resource settings, there is an abundance of overlapping credentials with the state such as youth peer advocate, family peer advocate, and certified recovery peer advocate which can be confusing for those looking to enter the peer support workforce.

Additionally, many certifications lack specialization, leaving individuals unprepared for the unique demands of their work environments:

*“It gives you very basic information, but none that you can really apply immediately. And depending on the setting you work in, sometimes you’re left to fend for yourself. Some of the credentials don’t have specializations. So, if I’m a youth care advocate and I don’t work in a mental health facility, how is that? How is this supposed to help me, right?”* (Key Informant, United States)

Although credentials are meant to signal that individuals have been vetted through a formal process, in practice, this vetting may not always ensure adequate readiness or quality: “it’s not always the process that we would hope it is.” (Key Informant, United States)

#### Supervision.

KII and FGD participants based in the US were more likely to prefer an experienced peer supporter as a supervisor rather than a clinician, though many had clinical supervisors instead. One participant described how “most peer supporters are supervised by clinical staff, especially if it’s Medicaid reimbursable and within a public mental health system” (Focus Group Participant, United States). Another FGD participant described how having a clinical supervisor was more challenging:

*“If you have a clinical supervisor, it makes it harder...having a peer specialist as a supervisor who identifies as someone with lived experience and has worked in the role (provides an) opportunity for connection.* (Focus Group Discussion Participant, United States)

Several KII participants outside of the US also described challenges with the clinical supervisor model, suggesting that it would not make sense for such distinct roles to supervise each other. One participant equated it to “having a clinician reporting to a senior peer worker.” (Key Informant, Australia)

Participants in the US described how peer supporters could become supervisors, for example: “all the state has to do is to say what qualifications that certified peers need to have in order to be a supervisor” (Key Informant, United States). Another participant described their experience of being a supervisor and helping others get certified to do so as well: “I follow up with people...and support them to get the training that they need and the time that they need to pass the test and then get certified (to be a supervisor).” (Key Informant, United States)

Outside of the US, KII and FGD participants were more likely to endorse a model of co-supervision. One KII participant stated:

*I think it has to be a team, and the team has to include a therapist and a peer support person. There has to be a professional point of view to ensure boundaries are not crossed and there are not any big mistakes that can occur because the peer support person doesn’t know everything about mental illness and mental issues.* (Key Informant, Israel)

Another participant added that: “I agree (peer supporters) are capable (of supervision), but there is a benefit of co-supervision - I mean the clinician and then also someone with peer experience.” (Key Informant, Singapore).

Supervision occurred in both group and one-on-one formats in which the peer supporters may share present challenges within their work and seek support. In addition to navigating challenges, both formats of supervision also created a space for peer supporters to seek emotional support for their own lived experience. One participant who currently acts as a supervisor also described that within their role they provide technical assistance to their peers in navigating and understanding the training, examination, and certification process. An additional participant also noted the importance of supervisors in enforcing role boundaries and minimizing the risk of peer drift.

#### Challenges and motivating factors to delivering peer support services.

Participants were asked in the survey if they experienced challenges in their time as a peer supporter, with 89.1% of participants reporting that they do. If a participant responded “yes” they were prompted with an open text field to elaborate on what specific challenges they faced. Further themes related to challenges and motivating factors for peer supporters were explored in the FGDs and KIIs, and included the following:

*Stigma, discrimination, and lack of respect:* Survey participants expressed that clinical colleagues, management, and community members may perpetuate stigmatizing perceptions of mental health challenges through discrimination, microaggressions, and tokenization. Participants also consistently reported common hierarchies they faced and the perception that they were “less professional” or “unqualified” due to their own lived experiences.

*Emotional toll, workplace safety, and psychosocial hazards:* Survey participants shared that they often face emotional exhaustion, burnout, and compassion fatigue. As persons in recovery, they also shared how certain workplace inadequacies, such as understaffing, lack of structural support, and lack of trauma-informed workplaces, made them more vulnerable to psychosocial hazards.

*Financial insecurity and limited career progression:* Participants repeatedly expressed widespread concern about low wages, lack of benefits, and compensation inequity relative to skill and responsibility. Participants also shared the lack of a clear peer-specific career progression pathway, as well as the lack of ongoing opportunities for professional development and/or certification that are respected by broader systems.

*Challenges in engaging with peers:* Survey participants occasionally shared additional challenges they faced when directly supporting their peers. These barriers included difficulties engaging with individuals’ internalized stigma, distrust in services, communication barriers, as well as supporting peers with comorbidities.

*Access to knowledge:* An external motivator acknowledged by one of the global participants was access to knowledge through the institution or organization that employed them as a peer supporter. They stated that their affiliation with their employer’s organization allowed them increased access to research through libraries or professors. Other participants also expressed a desire for more educational opportunities through their employment.

*Intrinsic motivation and desire to assist others in need:* The most common source of motivation cited by both US and global participants was that they are intrinsically motivated to help others in need. Participants felt that their work as peer supporters gave them a sense of meaning and purpose. They found that sharing their own experiences with their mental health was validating and that being able to be a resource for someone else who experienced similar challenges to them was rewarding. In some cases, participants said that being able to help others helped them be resilient in the face of many challenges, providing peer support and preventing burn-out.

## Discussion

Peer support is an effective strategy for improving wellbeing of those living with serious mental health challenges [16–18]. While many studies have addressed questions regarding the effectiveness of peer support services [32], much remains to be known about the experiences that peer supporters have in delivering services, participating in training and supervision, and navigating complex ecosystems with multiple stakeholders in a global context. The contribution of this study is to illuminate what peer supporters find meaningful about their roles and what barriers and facilitators may exist when delivering peer support services in diverse global contexts.

This study has important implications for future research and implementation of peer support services. In qualitative data, peer supporters provided many examples of their internal motivation to deliver peer support services. This included sharing their lived experience in the hope that it would help someone else in their recovery journey. However, multiple barriers affecting external motivation were noted in the survey, key informant interviews, and focus group discussions, such as lack of career pathways and equitable compensation. It was notable that respondents across settings described similar barriers, including settings where peer support is more standardized. This finding aligns with other studies describing barriers associated with the sustainment of peer support services [33,34]. Opportunities for professional growth and equitable compensation are associated with higher-quality service delivery in human services fields [35].

It is important that the skills and competencies that are reported as most widely used and valued in peer support service provision are embedded into peer support training. The most reported competencies in the survey were sharing lived experience of recovery, collaboration and care, and communication. Qualitative findings confirmed survey findings and provided examples of these skills in practice, and furthermore, defined what it means to be a “Peer” - someone whose positionality and shared experience is around their mental health challenge. In the subsequent key informant interviews and focus group discussions, participants elaborated on these competencies and described their role as knowing when and how to share their lived experience, being there to collaborate and support rather than to direct, and sharing information in a simple and comprehensive manner. We recommend that standardized sets of competencies be developed and utilized at national levels, which will create more transparency regarding the role of peer supporters and clarity in reporting quality outcomes. Furthermore, standardized competencies will protect the role of the peer and support diverse teams of mental health care providers to delineate between roles of clinicians and roles of peer supporters. For example, while skills like communication may be relevant regardless of the role, other skills such as knowing when and how to share one’s own lived experience to support another person’s recovery is unique to the role of the peer supporter.

Participants described frustrations with the training they had received, including insufficient length, lack of specialized training, and feeling ill-prepared after completing training. Organizations and systems implementing peer support services may consider how to effectively streamline the path that a peer supporter takes – from initial training into service delivery. Case-based learning that includes role plays may supplement the knowledge gained through the initial training [36]. Though this theme did not arise in the data, many states within the US require a number of internship hours prior to beginning service delivery [37]. Furthermore, trainings should be curated carefully, embedding the most relevant skills and competencies, tailoring the training format (hybrid, in-person, online) to the needs of the setting and considering the benefits and downsides of each model. Beyond quality training, implementation protocols are important for ensuring high-quality service provision, and organizations must be aware that implementation may not be a one-size-fits-all approach.

Additionally, it is important that peer supporters are trained to understand their scope and role, and that this is protected by their supervisors and affiliated organizations. In interviews, several participants described moments of role drift where they moved beyond what they understood to be their role and into more clinical or administrative roles. While the ecosystem of peer support services includes collaboration with clinicians, and this is a meaningful and effective partnership, most peers expressed a preference to be supervised by more experienced peers in addition to, or instead of, clinicians. It may be helpful for a wider range of peer supporters to have dedicated training in supervision, following the example provided by one key informant in the US. Our findings illuminated gaps in supervision – nearly half of survey respondents had experience in supervision, while only 62.5% of those had received training prior to becoming a supervisor. Qualitative findings expanded on challenges faced during supervision, pointing to a need for more qualified peer supporters to serve in these roles. Mentorship and support from another experienced peer supporter, in addition to meaningful chances to interact with others in mental health service provision roles, may help with reducing stigma, which was frequently reported as an ongoing issue both in the US and globally. Furthermore, many peer supporters described the emotional burden and burnout they experience within their roles, with the potential to have their own mental health challenges triggered and/or stigmatized. An experienced peer supporter is more equipped to support another peer supporter with potential triggers and emotional safety [38]. Both qualitative and quantitative findings confirmed a set of competencies that were described as useful within a peer support role, and these can be referenced by supervisors seeking to use peer supporters most effectively.

Our study revealed key differences in the implementation context across settings, and opportunities for meaningful partnership across the mental health ecosystem. Peer support services tended to be less standardized and regulated outside of the US, Canada, Denmark, and Australia. Even across states in the US, requirements for training and experiences of service delivery varied despite the existence of regulating national bodies like the Substance Abuse and Mental Health Services Administration. Several participants outside of the US, Canada, Denmark, and Australia provided examples of community organizations like faith communities stepping in to fill the gap to support peer support services. However, the lack of standardization also increased the risk of role drift or inequitable compensation. These findings highlight the importance of tailoring the implementation model to the local context and partnering with grassroots organizations. While integration of peer support services within grassroots organizations is becoming more common, it remains a relatively underutilized implementation strategy that can build trust and decrease stigma and increase access to care [39]. The ongoing UPSIDES trial provides an effective example of leveraging community resources to increase access to care [40]. In Kenya, local churches identified by communities as trusted institutions have been leveraged to deliver family mental health care and HIV prevention [41]. Similarly in Texas, faith-based communities are collaborating with the EMPOWER program to leverage their congregations to deliver evidence-based psychosocial interventions for people with depression [42,43].

Although peer supporters are considered specialist providers in many contexts, including the US where certification is mandated for medicaid reimbursement, the broader literature on task-sharing highlights effective strategies in expanding mental health care through community providers. Issues such as burnout, lack of career progression, financial renumeration, and the need for higher-quality training and supervision persist across community-based mental health care. Expanding cadres of non-clinician mental health service providers is critical for addressing the care gap, yet challenges with scalability and quality of delivery will persist without adequate resources and policies such as hybrid training models, regular supervision, and fair compensation [44]. To this end, Aves Mental Health, formally known as the Global Mental Health Peer Network, has published the Renumeration Framework and Inclusion Assessment Tool to guide organizations, working in global contexts, toward fair compensation and inclusion of peer supporters within the mental health ecosystem [45]. The implementation of peer support services in diverse settings will benefit from the broader literature on task-sharing and the importance of adapting implementation models to the local context. Thus, while some elements of training and supervision, such as the core competencies for peer support, may be globally applicable, these models must be tailored to local contexts, for example the feasibility of the deployment of digital platforms or the availability of formal peer support services [46].

### Limitations

This study had a small sample size of participants, recruited through convenience and snowball sampling methods, and therefore not representative of all service environments or global experiences. We leveraged professional networks for the convenience and snowball sampling of the survey respondents and focus group discussion participants. This sampling strategy may bias the results toward more well-connected participants and participants in high income settings leading to less representation of informal peers and peers without institutional ties. The sample of the survey participants in this study was biased towards peers located in the United States and identifying as white. Thus findings from this study may be less representative of experiences of peers from other communities and from the Global South. Additionally, many participants in the KIIs and FDGs were located in the United States, limiting the generalizability of the qualitative data. To reach Spanish-speaking peer supporters, we translated and distributed the survey in Spanish and worked with a partner to distribute the survey within Spanish-speaking peer networks. However, no survey respondents took the survey in Spanish. All data collection was conducted in English, thereby limiting our understanding of peer support services, particularly in countries where English is not predominantly spoken. The results of this study should therefore be interpreted cautiously when considering implications for peer support in global contexts.

## Conclusion

Peer support services are an effective model for supporting those with serious mental health challenges, yet training and implementation of peer support varies significantly within the US and globally. Future peer support trainings and implementation models may ensure that peer supporters are trained on the most relevant skills and competencies, that peers can receive supervision from others with lived/living experience of recovery and become supervisors themselves, and that training format and implementation protocols are tailored to the unique needs of the local context.

## Supporting information

S1 Text Survey Questions: The set of questions we included for our survey which aimed to capture quantitative and qualitative data on peer supporters experiences with training, service delivery, and supervision.
(PDF)

S1 Data Survey Participant Demographics: Summarized results for the demographic data collected from survey participants.
(PDF)

S2 Data Focus Group Discussion Participant Demographics: Summarized results for the demographic data collected from focus group discussion participants.
(PDF)
