# Supplementary material for: “Peer with a P versus a p”: A mixed-methods study of peer support training, service delivery, and supervision across global contexts
Source: PLOS Ment Health. 2026 Jan 12;3(1):e0000447. doi: 10.1371/journal.pmen.0000447 (PMC12798151; doi:10.1371/journal.pmen.0000447)
Supplement: S1 Text — The set of questions we included for our survey which aimed to capture quantitative and qualitative data on peer supporters experiences with training, service delivery, and supervision. (PDF) [file pmen.0000447.s001.pdf]

HARVARD
MEDICAL SCHOOL

Section A: Consent

Please Choose One of the Following/ Por Favor

- ○ To continue in English, press here
- ○ Para seguir en Español, hacer clic aquí

You are being asked to take part in a research study.

The research is being conducted to learn about your experience as a peer support worker. Specifically, we are interested in learning more about your role, the training you received, and the skills you use in providing support. You are being asked to participate in this research because of your experience as a peer support worker.

Your participation in this study is voluntary and you may withdraw your participation at any time for any reason.

If you take part in this study, you will be asked to complete

a brief questionnaire about you and your experience delivering peer support to those with mental health challenges. The questionnaire is expected to take approximately 10 minutes to complete. Additionally, you will have the opportunity to indicate interest in participating in a 90 minute focus group discussion. If eligible and selected to participate in the focus group discussion, you will be asked a series of questions regarding your experience receiving training, delivering peer support interventions, experience with supervision, and guidance on expanding access to peer support training.

Participant data, including your consent and questionnaire responses will be securely stored using a shared Microsoft OneDrive folder only the Harvard Medical School team has access to.

Potential risks for completing the questionnaire include:
* The identity of participants can be known to others if the questionnaire is taken in the presence of others, or in the case of a data breach.

There are no direct benefits to you from your participation in this questionnaire. We cannot promise any benefits to you or others from your participation in this questionnaire. However, possible long-term societal benefits include expanding access to peer support training, expanding

access to mental health services, and contributing to reducing stigma for those living with a mental health challenge.

You can decline to participate in any part of this study for any reason and can end your participation at any time. By providing consent below, you grant Harvard Medical School permission to share your data and use your response to inform future research. All data collected from this questionnaire will be de-identified, and your identity will remain confidential if you choose to participate in the study.

If you have questions about this study, you can contact Isaac Lara at empower@hms.harvard.edu

Thank you again for your time and participation.

Please indicate you are 18 years of age or older and understand the purpose of this survey and that you would like to proceed:

- ○ Yes
- ○ No

I consent to participate in the study:

- Yes
- No

I consent to the use of de-identifiable data in research publications and presentations:

- Yes
- No

Section B: Eligibility

Are you currently working as a peer support worker and delivering peer mental health support to those with mental health challenges who face difficulties with daily functioning?

- Yes
- No

Section C: Demographic Questions

Please provide the country you work in below

Please provide the state you work in below.

Please provide the city/town you work in.

How old are you?

	18	38	59	79	99
Age					

Please choose the gender identity that best describes you:

- ○ Cisgender Male
- ○ Cisgender Female
- ○ Transgender Male
- ○ Transgender Female
- ○ Non-binary/Non-conforming
- ○ Prefer not to say
- ○ [ ] Other, please specify

Please choose the sexual orientation that best describes you:

- ☐ Homosexual; Gay or Lesbian
- ☐ Bisexual
- ☐ Heterosexual or Straight
- ☐ Asexual
- ☐ Pansexual
- ☐ Queer
- ☐ Prefer not to say
- ☐ [ ] Other

Choose one or more races that you consider yourself to be:

- ○ White or Caucasian
- ○ Black or African American
- ○ American Indian/Native American or Alaska Native
- ○ Asian
- ○ Native Hawaiian or Other Pacific Islander
- ○ Prefer not to say
- ○ [ ] Other

Are you of Hispanic, Latino, or Spanish origin?

- ○ Yes
- ○ No
- ○ Prefer not to say

What is the highest degree or level of education you have completed?

- ○ Some high school or less
- ○ High school diploma or GED
- ○ Some college, but no degree
- ○ Associates or technical degree
- ○ Bachelor's degree

- ◯ Graduate degree (MA, MS, MBA, etc.)
- ◯ Professional degree (PhD, JD, MD, DDS, etc.)
- ◯ Prefer not to say

Section D: Peer Support Training & Certification

In what country did you receive peer support training?

Please describe the state/province in which you received peer support training.

Please write below the name of the organization that provided peer support training

What was the modality of your peer support training?

- Virtual or Online
- In-Person
- Hybrid (A combination of virtual and in-person)

Were there any financial costs associated with training and/or certification?

- Yes
- No

What was the total cost to be trained and certified?

Were there opportunities to apply for scholarships to cover or reduce the associated costs?

- Yes
- No

Did you receive certification for peer support?

- Yes
- No

Please select the requirements you had to fulfill in order to receive certification:

- Training
- Standard Examination
- Internship/Practice

How many hours of training were you required to complete

0 12 24 36 48 60 72 84 96 108 120

Time

How many hours of internship/practice were you required to complete

0 20 40 60 80 100 120 140 160 180 200

Time

Section E: Current Work

What percent of your time involves delivering peer support to those with mental health challenges?

	0	10	20	30	40	50	60	70	80	90	100
Percent											

Please select the peer support techniques which you use in providing support to those with mental health challenges.

- [ ] Collaboration and care
- [ ] Mutual support
- [ ] Sharing lived experience of recovery
- [ ] Personalization
- [ ] Recovery planning
- [ ] Resource sharing
- [ ] Skill teaching
- [ ] Crisis management
- [ ] Communication
- [ ] Teamwork
- [ ] Leadership and advocacy
- [ ] Growth and development

Please select the clinical techniques which you use in providing support to those with mental health challenges.

- ☐ Mental health diagnosis and pscyhoeducation
- ☐ Cognitive behavioral techniques
- ☐ Basic counseling skills
- ☐ Acceptance and commitment therapy
- ☐ Case management

Please list any techniques not listed above that you regularly use to deliver peer support to those with mental health challenges.

Please indicate the number of years you have delivered peer support to those with mental health challenges.

0 10 20 30 40 50 60

Number of years

Do you experience any challenges working in the field as a peer support worker?

- Yes
- No

Please describe the challenge(s) you experience.

Section F: Peer Supervision

Do you currently work or have you previously worked as a direct supervisor to peer support workers?

- Yes
- No

What percent of your time involves supervising peer support workers?

0 10 20 30 40 50 60 70 80 90 100

Percent

Please select the supervision format(s) you use within your role

- [ ] Group meetings
- [ ] One-on-one sessions
- [ ] Documented reports
- [ ] Other

Please indicate the number of years you worked as a supervisor to peer support workers

0 10 20 30 40 50 60

Number of years

Did you receive additional training in order to supervise peer support workers?

- Yes
- No

Please list the training program you completed to become a peer supervsior

How many peer support workers do you supervise at a time?

Please tell us about your experience with your supervisor

Section G: Referral

If you would like to refer others with direct peer support experience to complete this survey, please include their email contact information below:

Section H: FGD Interest Form

In addition to this survey, our team plans on conducting Focus Group Discussions with peer support workers to explore their current experience with providing peer support to those with mental health challenges that impact daily functioning. We hope to learn more about peer support workers' role, the training received, and the skills used in

providing support. We are developing a standardized, open access, digital peer support training curriculum to expand the peer workforce, both nationally and globally. We are also interested in gathering peer support workers' thoughts and feedback about the content of such a mental health intervention.

If you are interested in participating in this focus group discussion, please provide your contact information below so that a member of the research team can contact you to discuss your potential participation. No one outside the research team will have access to the information you provide. Participants selected to participate in the focus group discussion are eligible to receive a $50 Amazon gift card.

Please note: indicating interest in participation of the focus group discussion does not guarantee involvement. Only participants selected and involved in the focus group discussions will receive the Amazon gift card

Are you interested in participating in a focus group discussion?

- ○ Yes

○ No

Please provide your name *(this information will only be shared with the research team, and will be used for the purpose of identifying you if selected to participate in a focus group discussion)*

Please provide your email address

Please provide your phone number, including country code

Sección A: Consentimiento

Se le pide que participe en un estudio de investigación.

La investigación se lleva a cabo para conocer su experiencia como trabajador de apoyo entre pares (peer support worker, por sus siglas en inglés). En concreto, estamos interesados en saber más sobre los roles o funciones que tuvo, la formación que ha recibido y las habilidades que utiliza para proporcionar apoyo. Se le pide que participe en esta investigación por su experiencia como trabajador de apoyo entre pares.

Su participación en este estudio es voluntaria y puede retirarla en cualquier momento y por cualquier motivo.

Si participa en este estudio, se le pedirá que rellene un breve cuestionario sobre usted y su experiencia prestando apoyo entre iguales a personas con problemas de salud mental. El cuestionario le llevará aproximadamente 10 minutos. Además, tendrá la oportunidad de indicar su interés en participar en un grupo de discusión de 90 minutos. Si reúne los requisitos y es seleccionado para participar en el grupo de discusión, se le harán una serie de preguntas sobre su experiencia recibiendo formación, realizando intervenciones de apoyo entre pares, experiencia con la supervisión y orientación sobre cómo ampliar el acceso a la formación de apoyo entre iguales.

Los datos de los participantes, incluido su consentimiento y las respuestas al cuestionario, se almacenarán de forma segura en una carpeta compartida de Microsoft OneDrive a la que sólo tendrá acceso el equipo de la Facultad de Medicina de Harvard.

Los riesgos potenciales de completar el cuestionario incluyen: * La identidad de los participantes puede ser conocida por otras personas si el cuestionario se realiza en presencia de otras personas, o en el caso de una violación de datos.

Su participación en este cuestionario no le reportará ningún beneficio directo. No podemos prometerle ningún beneficio a usted o a otras personas por su participación en este cuestionario. Sin embargo, los posibles beneficios sociales a largo plazo incluyen la ampliación del acceso a la formación de apoyo entre pares, la ampliación del acceso a los servicios de salud mental y la contribución a la reducción del estigma de las personas que viven con algún problema de salud mental.

Puede negarse a participar en cualquier parte de este estudio por cualquier motivo y puede poner fin a su participación en cualquier momento. Al dar su consentimiento, autoriza a la Escuela de Medicina de

Harvard a compartir sus datos y a utilizar sus respuestas para futuras investigaciones. Todos los datos recogidos de este cuestionario serán des-identificados, y su identidad permanecerá confidencial si decide participar en el estudio.

Si tiene alguna pregunta sobre este estudio, puede ponerse en contacto con Isaac Lara en empower@hms.harvard.edu.

Gracias de nuevo por su tiempo y su participación.

Por favor, indique que tiene 18 años o más, que comprende el propósito de esta encuesta y que desea continuar:

- Sí
- No

Doy mi consentimiento para participar en el estudio:

- Sí
- No

Doy mi consentimiento para que se utilicen datos no identificables en publicaciones y presentaciones de investigación:

- Sí
- No

Sección B: Admisibilidad

¿Trabaja actualmente como trabajador de apoyo entre pares (peer support) y presta apoyo en salud mental a personas con problemas de salud mental que tienen dificultades para desenvolverse en la vida diaria?

- Sí
- No

Sección C: Preguntas demográficas

Indique a continuación el país en el que trabaja

Indique a continuación el estado en el que trabaja.

Indique la ciudad/localidad en la que trabaja.

¿Cuántos años tiene?

18 38 59 79 99

Edad ()

Por favor, elija la identidad de género que mejor le describa:

- Hombre cisgénero
- Mujer cisgénero
- Hombre transexual
- Mujer transexual
- No binario/no conforme
- Prefiero no decirlo

- [ ] Otros, especifique

Por favor, elija la orientación sexual que mejor le describa:

- Homosexual; Gay o lesbiana
- Bisexuales
- Heterosexual o Heterosexual
- Asexual
- Pansexual
- Queer
- Prefiero no decirlo
- [ ] Otros

Elige una o más razas de las que te consideras:

- Blanco o caucásico
- Negro o afroamericano
- Indio Americano/Nativo Americano o Nativo de Alaska
- Asiáticos
- Nativo de Hawai u otras islas del Pacífico
- Prefiero no decirlo
- [ ] Otros

¿Es usted de origen hispano, latino o español?

- Sí
- No
- Prefiero no decirlo

¿Cuál es el título o nivel de estudios más alto que ha completado?

- Algunos estudios secundarios o menos
- Bachillerato o GED
- Algunos estudios universitarios, pero sin titulación
- Diplomado o técnico universitario
- Licenciatura
- Título de posgrado (MA, MS, MBA, etc.)
- Grado doctoral (PhD, JD, MD, DDS, etc.)
- Prefiero no decirlo

Sección D: Formación y certificación de apoyo entre iguales

¿En qué país recibiste formación sobre apoyo entre pares?

Por favor, describa el estado/provincia en el que recibió la formación de apoyo entre pares.

Por favor, escriba a continuación el nombre de la organización que impartió la formación de apoyo entre pares

¿Cuál fue la modalidad de tu formación de apoyo entre pares?

- Virtual o en línea
- Presencial
- Híbrido (Una combinación de virtual y presencial)

¿Hubo costes financieros asociados a la formación y/o certificación?

- Sí
- No

¿Cuál fue el coste total de la formación y certificación?

¿Hubo oportunidades de solicitar becas para cubrir o reducir los costes asociados?

- Sí
- No

¿Recibió una certificación para el apoyo entre pares?

- Sí
- No

Seleccione los requisitos que tuvo que cumplir para recibir la certificación:

- Formación o entrenamiento
- Examen estandarizado
- Prácticas o rotaciones

¿Cuántas horas de formación se le pidieron completar?

	0	12	24	36	48	60	72	84	96	108	120
Tiempo ()											

¿Cuántas horas de prácticas tuvo que realizar?

	0	12	24	36	48	60	72	84	96	108	120
Click to write Choice 1											

Sección E: Trabajo actual

¿Qué porcentaje de su tiempo dedica a ofrecer apoyo entre pares a personas con problemas de salud mental?

	0	10	20	30	40	50	60	70	80	90	100	
Porcentaje ()												

Por favor, seleccione las técnicas de apoyo entre pares que utiliza para ayudar a las personas con problemas de salud mental.

- Colaboración y atención
- Apoyo mutuo
- Compartir experiencia personal de recuperación
- Personalización
- Planificación de la recuperación
- Reparto de recursos
- Enseñanza de habilidades o técnicas
- Manejo o gestion de crisis
- Comunicación
- Trabajo en equipo
- Liderazgo y defensa
- Crecimiento y desarrollo

Por favor, seleccione las técnicas clínicas que utiliza para ayudar a las personas con problemas de salud mental.

- Diagnóstico de salud mental y psicoeducación
- Técnicas cognitivo-conductuales
- Habilidades básicas de asesoramiento (counseling)
- Terapia de aceptación y compromiso
- Gestion o manejo de casos

Por favor, enumere cualquier técnica no mencionada anteriormente que utilice habitualmente para ofrecer apoyo entre pares a personas con problemas de salud mental.

Indique el número de años que lleva prestando apoyo entre iguales a personas con problemas de salud mental.

	0	6	12	18	24	30	36	42	48	54	60
Número de años ()											

¿Experimenta algún reto en el trabajo de campo como proveedor de apoyo entre pares?

- Sí
- No

Describa los retos que experimenta.

Sección F: Supervisión entre iguales

¿Trabaja actualmente o ha trabajado anteriormente como supervisor directo de trabajadores de apoyo entre pares?

- Sí
- No

¿Qué porcentaje de su tiempo dedica a supervisar a los trabajadores de apoyo entre pares?

0 10 20 30 40 50 60 70 80 90 100

Porcentaje ()

Seleccione el formato o formatos de supervisión que utiliza en su función

- Reuniones de grupo
- Sesiones individuales
- Informes documentados
- Otros

Por favor, indique el número de años que ha trabajado como supervisor de trabajadores de apoyo entre pares

0 6 12 18 24 30 36 42 48 54 60

Número de años ()

¿Recibió formación adicional para supervisar a los trabajadores de apoyo entre pares?

- Sí

○ No

Por favor, indique el programa de formación que ha realizado para convertirse en supervisor.

¿A cuántos trabajadores de apoyo entre pares supervisa a la vez?

Háblenos de su experiencia con su supervisor

Sección G: Remisión

Si desea remitir a otras personas con experiencia directa en el apoyo entre pares para que completen esta

encuesta, incluya a continuación sus datos de contacto por correo electrónico:

Sección H: Formulario de interés del DGF

Además de esta encuesta, nuestro equipo tiene previsto llevar a cabo grupos de discusión con los trabajadores de apoyo para explorar su experiencia actual en la prestación de apoyo a las personas con problemas de salud mental que afectan a su funcionamiento diario. Esperamos aprender más sobre el papel de los trabajadores de apoyo entre pares, la formación recibida y las habilidades utilizadas para proporcionar apoyo. Estamos desarrollando un plan de estudios estandarizado, digital y de libre acceso para ampliar el personal de apoyo entre pares, tanto a nivel nacional como mundial. También estamos interesados en recoger las opiniones y comentarios de los trabajadores de apoyo entre pares sobre el contenido de una intervención de salud mental de este tipo.

Si está interesado en participar en este grupo de discusión, por favor proporcione su información de contacto a continuación para que un miembro del equipo de

investigación pueda ponerse en contacto con usted para discutir su posible participación. Nadie ajeno al equipo de investigación tendrá acceso a la información que facilite. Los participantes seleccionados para participar en el grupo de discusión podrán recibir una tarjeta regalo de 50 dólares de Amazon.

Tenga en cuenta que indicar su interés en participar en el grupo de discusión no garantiza su participación. Sólo los participantes seleccionados y que participen en los grupos de discusión recibirán la tarjeta regalo de Amazon.

¿Está interesado en participar en un grupo de discusión?

- ○ Sí
- ○ No

Por favor, indique su nombre (esta información sólo se compartirá con el equipo de investigación y se utilizará para identificarle si es seleccionado para participar en un grupo de discusión)

Indique su dirección de correo electrónico

Indique su número de teléfono, incluido el codigo del país.

Powered by Qualtrics
