# Supplementary material for: “Peer with a P versus a p”: A mixed-methods study of peer support training, service delivery, and supervision across global contexts
Source: PLOS Ment Health. 2026 Jan 12;3(1):e0000447. doi: 10.1371/journal.pmen.0000447 (PMC12798151; doi:10.1371/journal.pmen.0000447)
Supplement: S1 Data — Summarized results for the demographic data collected from survey participants. (PDF) [file pmen.0000447.s002.pdf]

S1 Data. Survey Demographics

Variable	Total Sample (N = 101)	US Participants (n = 57)	Non-US Participants (n = 33)
Country (n, %)			
Algeria	1 (1)		1 (1)
Australia	7 (6.9)		7 (6.9)
Colombia	1 (1)		1 (1)
Denmark	6 (5.9)		6 (5.9)
Ethiopia	1 (1)		1 (1)
Hong Kong (S.A.R.)	1 (1)		1 (1)
Indonesia	1 (1)		1 (1)
Kenya	7 (6.9)		7 (6.9)
Nigeria	2 (2)		2 (2)
Pakistan	1 (1)		1 (1)
South Africa	1 (1)		1 (1)
Uganda	2 (2)		2 (2)
United Kingdom of Great Britain and Northern Ireland	1 (1)		1 (1)
United States	57 (56.4)	57 (56.4)	
Viet Nam	1 (1)		1 (1)
Does not state	11 (10.9)		
Average age in years (Range, SD)	45 (24-78, 14.0)	48.6 (24-78, 14.3)	40.3 (24-67, 12.1)
Gender (n, %)			
Cisgender Male	35 (34.7)	17 (16.8)	18 (17.8)
Cisgender Female	45 (44.6)	26 (25.7)	19 (18.8)
Non-binary/non-conforming	6 (6.0)	5 (5)	1 (1)
Transgender Male	2 (2)	2 (2)	
Other	5 (5)	1 (1)	4 (4)
Prefer not to say	5 (5)	3 (3)	2 (2)
Sexual Orientation (n, %)			
Heterosexual or straight	74 (73.3)	42 (41.6)	32 (31.7)
Homosexual; Gay or Lesbian	3 (3)	1 (1)	2 (2)

Queer	5 (5)	4 (4)	1 (1)
Asexual	5 (5)	1 (1)	2 (2)
Bisexual	8 (7.9)	5 (5)	3 (3)
Pansexual	2 (2)	1 (1)	1 (1)
Other	1 (1)		1 (1)
Prefer not to say	5 (5)	2 (2)	3 (3)
Race (n, %)			
Black or African American	21 (20.8)	5 (5)	16 (15.8)
White or Caucasian	66 (65.3)	46 (45.5)	20 (19.8)
Asian	4 (4)	1 (1)	3 (3)
American Indian/Native American or Alaska Native	2 (2)	1 (1)	1 (1)
Other	5 (5)	2 (2)	3 (3)
Prefer not to say	3 (3)	1 (1)	2 (2)
Hispanic or Latino Origin (n, %)			
Yes	10 (10)	9 (8.9)	1 (1)
No	87 (86.1)	45 (44.6)	42 (41.6)
Prefer not to say	2 (2)	1 (1)	1 (1)
Education (n, %)			
Some high school or less	3 (3)	1 (1)	2 (2)
High school diploma or GED	5 (5)	2 (2)	3 (3)
Associates or technical degree	18 (17.8)	12 (11.9)	6 (5.9)
Some college, but no degree	14 (13.9)	12 (11.9)	2 (2)
Bachelor's degree	38 (37.6)	19 (18.8)	19 (18.8)
Graduate degree (MA, MS, MBA, etc.)	18 (17.8)	9 (8.9)	9 (8.9)
Professional Degree (PhD, JD, MD, DDS, etc.)	4 (4)	1 (1)	3 (3)
Prefer not to say	1 (1)		1 (1)

Average number of years as a peer supporter (Range, SD)	7.8 (1-33, 7.4)	9.9 (1-33, 8.4)	5 (1-23, 4.7)
