# Supplementary material for: “Peer with a P versus a p”: A mixed-methods study of peer support training, service delivery, and supervision across global contexts
Source: PLOS Ment Health. 2026 Jan 12;3(1):e0000447. doi: 10.1371/journal.pmen.0000447 (PMC12798151; doi:10.1371/journal.pmen.0000447)
Supplement: S2 Data — Summarized results for the demographic data collected from focus group discussion participants. (PDF) [file pmen.0000447.s003.pdf]

S2 Data. Focus Group Discussion Demographics

Variable	Total Sample (N = 14)	US Participants (n = 7)	Non-US Participants (n = 6)
Country (n, %)			
Indonesia	1 (7.1)		1 (7.1)
Kenya	1 (7.1)		1 (7.1)
Nigeria	1 (7.1)		1 (7.1)
South Africa	1 (7.1)		1 (7.1)
Uganda	1 (7.1)		1 (7.1)
United States	7 (50)	7 (50)	
Viet Nam	1 (7.1)		1 (7.1)
Does not state	1 (7.1)		
Average age in years (Range, SD)	42.1 (24-63, 13.3)	44.9 (31-62, 13.2)	39.3 (24-63, 13.8)
Gender (n, %)			
Cisgender Male	7 (50)	4 (28.6)	3 (21.4)
Cisgender Female	5 (35.7)	2 (14.3)	3 (21.4)
Non-binary/non-conforming	1 (7.1)	1 (7.1)	
Other	1 (7.1)		1 (7.1)
Sexual Orientation (n, %)			
Heterosexual or straight	7 (50)	4 (28.6)	3 (21.4)
Homosexual; Gay or Lesbian	1 (7.1)		1 (7.1)
Queer	2 (14.3)	1 (7.1)	1 (7.1)
Bisexual	1 (7.1)	1 (7.1)	
Prefer not to say	3 (21.4)	1 (7.1)	2 (14.3)
Race (n, %)			
Black or African American	3 (21.4)	0 (0)	3 (21.4)
White or Caucasian	6 (42.9)	6 (42.9)	
Asian	3 (21.4)	1 (7.1)	2 14.3)
Other	1 (7.1)		1 (7.1)
Prefer not to say	1 (7.1)		1 (7.1)

Hispanic or Latino Origin (n, %)			
No	14 (100)	7 (50)	7 (50)
Education (n, %)			
High school diploma or GED	2 (14.3)		2 (14.3)
Associates or technical degree	3 (21.4)	2 (14.3)	1 (7.1)
Some college, but no degree	2 (14.3)	2 (14.3)	0 (0)
Bachelor's degree	5 (35.7)	2 (14.3)	3 (21.4)
Graduate degree (MA, MS, MBA, etc.)	1 (7.1)	1 (7.1)	
Professional Degree (PhD, JD, MD, DDS, etc.)	1 (7.1)		1 (7.1)
Average number of years as a peer supporter (Range, SD)	4.9 (2-15, 3.6)	5.7 (2-15, 4.4)	4 (2-7, 2.4)
